# The distribution of the common mental disorders: social inequalities in Europe

**DOI:** 10.1186/1745-0179-1-14

**Published:** 2005-09-05

**Authors:** Tom Fryers, David Melzer, Rachel Jenkins, Traolach Brugha

**Affiliations:** 1 Psychiatry, University of Leicester, Leicester, UK; 2 Epidemiology and Public Health, University of Exeter, Exeter, UK; 3 WHO Collaborating Centre, Institute of Psychiatry, London, UK; 4 Psychiatry, University of Leicester, Leicester, UK; 5 International and Public Health, New York Medical College, Valhalla, USA

## Abstract

**Background:**

The social class distribution of the common mental disorders (mostly anxiety and/or depression) has been in doubt until recently. This paper reviews the evidence of associations between the prevalence of the common mental disorders in adults of working age and markers of socio-economic disadvantage.

**Methods:**

Work is reviewed which brings together major population surveys from the last 25 years, together with work trawling for all European population studies. Data from more recent studies is examined, analysed and discussed. Because of differences in methods, instruments and analyses, little can be compared precsiely, but internal associations can be examined.

**Findings:**

People of lower socio-economic status, however measured, are disadvantaged, and this includes higher frequencies of the conditions now called the 'common mental disorders' (mostly non-psychotic depression and anxiety, either separately or together). In European and similar developed populations, relatively high frequencies are associated with poor education, material disadvantage and unemployment.

**Conclusion:**

The large contribution of the common mental disorders to morbidity and disability, and the social consequences in working age adults would justify substantial priority being given to addressing mental health inequalities, and deprivation in general, within national and European social and economic policy.

## Introduction

This paper seeks to explore what is known about the associations of psychiatric disorders with indicators of social disadvantage, and therefore about social risk factors in individuals and populations, and the potential for targetting with additional resources to preventive or ameliorative ends.

The recent European Mental Health Status Project [1], commissioned by the European Commission, reviewed the data available on prevalence of mental illness in European populations in relation to social, economic and service factors. In this context, a 'Survey of Surveys' identified and collated over 200 population studies, but the methods of data collection, instruments, analytical methods, and presentation of results varied so much, that very few data were strictly comparable, and very limited meta-analysis proved possible [2].

However, in respect of social disadvantage and the distribution of psychiatric disorder, we were fortunate in having a recently completed review of the world literature, together with an extended analysis of the First British National Psychiatric Household Survey undertaken for the Government of the United Kingdom [3-6]. This paper first briefly summarises these two studies, adds data from a major German study published more recently, and from a number of smaller studies identified by the Survey of Surveys, and then considers some of the major issues arising from the results.

In all western countries, most physical diseases, and severe, 'psychotic' psychiatric disorders are well known to be distributed unequally by social position [7,8]. Psychotic disorders severely affect individual patients and their families, but are relatively rare. A far more extensive burden of mental illness in the community arises from less severe but more numerous 'common mental disorders', (often called 'neurotic illness'; mostly anxiety and depression, separately or together) for which associations with social position have been unclear in the scientific literature [9]. That, then is the focus of this paper.

## A systematic literature review; large-scale population studies

*(Fryers, Melzer & Jenkins, 2003 *[3]; *and Melzer, Fryers & Jenkins, 2004 *[6]

Before about 1980, population surveys had no validated, systematic instruments to identify psychiatric disorders, but several have been developed since. Measurement and classification of both mental disorder and social position carry inherent ambiguities; confidence in analysis and interpretation therefore require individual linked data in large populations. The following criteria were used to identify studies for inclusion in the review:

• community based studies (general household populations)

• populations encompassing a broad spectrum of social class variation

• samples of 3,000 or more adults of working age

• methods of identification of mental illness by validated standard instruments

• social position identified by explicit, standard markers

• a diagnostic range encompassing the common mental disorders

• individual data linking mental health measures and social indicators; i.e. not area studies

• relevance to UK policy development; studies from established market economies

• fieldwork undertaken since 1980

• published output on the key areas of interest

Computer-accessed research-literature data-bases were exhaustively searched, but they are often ineffective for broad or ambiguous categories, and proved so in this case. Moreover, they do not include books, or reports from research institutes or government departments, a necessary source of detailed information on large-scale population surveys. Most information came from cross references and direct enquiry of researchers and units, to create a unique database of almost 1,000 references.

Nine large-scale studies were identified which fulfilled the criteria (Table 1). For these, the published work was examined independently by two researchers, with regard to the validity and reliability of their methods, and their findings relating to the prevalence of the common mental disorders and differentials in social position. Of five European studies, four were from the UK and one from The Netherlands. One each was from Canada and Australia; two were from the USA. Since this work was finished, data have become available from the German National Health Survey of 1999 which appears to fulfil the inclusion criteria. This is described later.

**Table 1:** Surveys included in the Inequalities Review [6]

Annual Health Surveys for England (HSE), annually from 1993
National Psychiatric Morbidity Survey of Great Britain (household sample), 1993
Health and Life-style Survey (HLS), 1984–85 and follow-up, 1991–92
British Household Panel Survey (BHPS),1991–92
Netherlands Mental Health Survey and Incidence Study (NEMESIS), 1996

Edmonton Survey of Psychiatric Disorders (Canada), 1983–86
Australian National Survey, 1997
USA Epidemiologic Catchment Area Program (ECA), 1980–83
USA National Co-morbidity Study (NCS), 1990–92

Although all 9 studies used recognised instruments with at least some published validation, several different instruments were in use, and even the same instruments were applied in different ways. Categories of disorder, indicators of social position, and presentation of statistics were so diverse that no numerical meta-analysis was possible. Response rates, not always high, (54% – 80%) also prejudiced interpretation.

Poverty, education, housing, occupation, employment, social status and social engagement are relatively tangible measures, for which 'Social Class' or 'Socio-Economic Status' are merely proxies, but these markers of social disadvantage are not independent of each other. Other factors are known to be important – childhood experience, physical illness, life events, working situations, and social networks – but they were barely acknowledged by these large-scale cross-sectional studies. If we wish to have evidence of the direction of causation for associations discovered, we need longitudinal studies. The evidence available from the UK birth cohort studies is briefly summarised below and is available in more detail in the source documents. [6]

Nevertheless, some comparison of the cross-sectional studies was possible. In each study, the categories which most nearly approximated to the 'common mental disorders' were examined; usually this meant 'all affective disorders', 'all depressive disorders', 'dysthymia', and 'all anxiety disorders'. Similarly, in most studies, three indicators of social disadvantage could be compared: education, employment, and material circumstances, as well as occupational social status. Although the studies used different taxonomies, differentials within the taxonomy could be recorded for each one.

For education, the highest and lowest groups were compared, whether measured by years of education or qualifications achieved. For employment the 'unemployed and seeking work' were compared with either 'all others of working age', or 'all employed'. Material circumstances were measured in many ways, but the lowest and highest in each hierarchy could be compared. The associations detected were subjected to statistical tests of significance, and odds ratios for each relationship quoted wherever possible.

Taking higher prevalence of disorder in less privileged groups as a 'positive' association, of the nine population-based studies with adequate measures of mental health and indicators of social disadvantage, eight provided evidence of an association between less privileged social position and higher prevalence of the common mental disorders, on at least one of the available indicators (Table 2). The one study showing no clear relationships had the lowest response rate (54%), which may have limited its capacity to demonstrate associations. Less education was 'positive' in four out of five studies. Unemployment showed positive associations in six out of seven studies, though in one study the association was positive only for men. Low income, wealth, assets, or other markers of material standard of living were positive in all six studies. Less privileged occupational social class was positive in three studies out of six. Perhaps most importantly, no study showed a contrary trend with any indicator.

**Table 2:** Number of included studies reporting associations with higher rates of the common mental disorders, by indicators of less privileged social position [3;6]

		*Less education*	*Unemployment*	*Lower income or material circumstances*	*Low social status*
Number of studies reporting associations	Total reporting	5	7	6	6
Positive	Men & women separately	2	3*	2	2
association	Men & women combined (separate data not given)	2	3	4	1
	Total positive	4	6	6	3
No clear association		1	1	0	3
Inverse association		0	0	0	0

Note: *one study, positive only for men; women equivocal.

These statistically significant positive associations do not reveal the degree of difference; compared to the most privileged groups, the most deprived groups seldom had more than a doubling in prevalence, that is odds ratios were almost always less than 2.

This simple overview suggests some robustness of findings despite the serious methodological limitations in reviewing such diverse studies. Education, employment and material circumstances provided better indicators than occupational social class, but there is remarkable consistency in the broad evidence from these nine large-scale population-based studies; the common mental disorders are significantly more frequent in socially disadvantaged populations.

## Limiting & disabling neurotic illness and markers of social disadvantage

*(Melzer, Fryers T, Jenkins R, Brugha T, & McWilliams B, 2003 *[4]; *and Melzer, Fryers & Jenkins, 2004 *[6]

Data from the 1993 National Psychiatric Survey of Great Britain [5] (supplied by the Data Archive, University of Essex) were subjected to detailed analysis:

• to clarify if markers of social position were independent of each other,

• to incorporate measures of disability into case identification,

• to indicate priority groups,

• to estimate effect sizes.

The 1993 Survey interviewed a representative household sample of over 10,000 people aged 16 to 64 using the Clinical Interview Schedule (CIS Revised) to record 'neurotic' symptoms or illness during the previous week. 15.5% had 'neurotic illness' which would justify clinical monitoring or active treatment in primary care; 63% of these had symptoms with an average duration of six months or more. Those reporting that "their mental symptoms stopped them doing things" were defined as having *'limiting neurotic disorder'*; those reporting also that they "had difficulty in doing at least one activity of daily living" were defined as having *'disabling neurotic disorder'*. In all groups most people had anxiety and/or depressive disorders.

Of the whole survey population, 8.3% had *'limiting neurotic disorder' *and 3.4% had *'disabling neurotic disorder'*. Consistent with the WHO Global Burden of Disease estimates [10], neurotic illness made a large contribution to all disability reported in the British survey. For example, of those with difficulties in three or more activities of daily living, 38% had a *'limiting neurotic disorder'*. Women had more neurotic illness than men, but risks were equal for *'disabling neurotic disorder'*.

Higher prevalence rates of the common mental disorders were associated with every marker of less privileged social position incorporated into the interview schedules, but multivariate analysis adjusting for gender, age and competing markers, left three as 'surviving independent markers':

• being unemployed or economically inactive

• poorer material circumstances (housing tenure and lack of car ownership)

• less education (having left full-time schooling before age 16)

Occupational social class was not a significant marker after adjustment.

*'Disabling neurotic disorder' *was associated with being economically inactive or unemployed (OR >2). In other analyses, *'disabling neurotic disorder' *was associated with having two or more physical illnesses (OR >6) and having two or more adverse life events (OR >3). Using other data from the survey, lone parents, those with physical diseases involving two or more disease systems, and those who were unemployed, together made up 20% of the population, but contributed 51% of those with *'disabling neurotic disorder'*.

Cross sectional data cannot clarify the direction of causation, though wider evidence provides some support for deprived circumstances causing the disorders [6]. Clarification needs longitudinal studies, which should include other potential risks, such as carer status, known to be associated with high rates of depression, and history of abuse. The lone parent group should receive special attention because of effects on the children.

## The European Survey of Surveys, 2002

The 'Survey of Surveys' of the European Mental Health Status Project identified more than 200 population surveys across Europe, but few provided comparable data because of differences in methods, instruments, analysis and presentation, or because they were small-scale community studies. A very restricted meta-analysis proved possible with surveys using a GHQ or CIDI instrument [2], including four of the five European studies in the review summarised above. These five are listed in Table 3 and briefly described below. Added to them is the German Health Survey of 1999 which appears to fulfil the same inclusion criteria as the studies reviewed, but has not yet published many results [11,12]

**Table 3:** Characteristics of European studies included in Maudsley review [6] with the German Health Survey, 1999 [11]. (Adapted from [6])

	European Surveys	Year	Type of study	Population sampled	Size of sample (achieved)	Response rate	Mental health instrument
**1**	Annual Health Surveys for England	1993, repeated annually	population survey	All adults in England, children from 1995	16,569 (1993)	76% for full interview, 66% for nurse tests (1993)	GHQ-12, cut-off 4+
**2**	National Psychiatric Morbidity Survey of Great Britain (household sample)	1993	population survey	All adults in England, Wales and Scotland (excluding Highland and Islands)	10,108	80%	Clinical Interview Schedule (CIS revised)
**3a**	Health and Life-style Survey	1984–85	population survey	Adults 18+, England, Wales, Scotland	9,003	73% for interview, 54% for self-completed questionnaire	GHQ-30 (+ a malaise measure)
**3b**	Health and Life-style Survey – follow-up	1991–92	follow-up of 84/85 respondents	Adults 18+, England, Wales, Scotland	5,352	59% of those interviewed in 1984/5 were re-interviewed	GHQ-30 (+ a malaise measure)
**4**	British Household Panel Survey	1991–92	population survey, with follow-up after one year	Adults aged 16+, households in Great Britain, south of Caledonian Canal	10,264	74% of 7,488 households	GHQ-12, cut-off 3+
**5**	Netherlands Mental Health Survey and Incidence Study (NEMESIS)	1996	population survey with follow-up at one and three years	Adults 18–64 resident in The Netherlands	7,147	64%	Composite International Diagnostic Interview (CIDI); GHQ-12
**6**	National German Health Survey (GHS)	1999	population survey	Adults 18–65 resident in Germany	4181	?	Composite International Diagnostic Interview (CIDI – Munich version)

### Health Survey for England, annually from 1993

Annually since 1991, adults aged 16 and over in England have been sample surveyed using structured interviews and clinical tests. Since 1993 most years have included the General Health Questionnaire (GHQ -12) [13], two questions about stress, and questions on perceived social support, occupation, income, material standard of living, and employment. Completed interviews have been approximately 16,000, a response rate of 74% of sampled households, and 92% of adults within these households [14,15].

A 'positive' score (4 or more on the GHQ -12), was considered to indicate a psychiatric disorder diagnosable by a clinician. Year by year there has been little variation in results; in 1998 for example, 13% of men and 18% of women were recorded as 'positive', correlated highly with perceived lack of social support, recent acute sickness, and long-standing illness. There were weak associations with occupational social class, but significant and progressive associations with low 'equivalised household income', especially among men (9% in the highest income quintile to 20% in the lowest income quintile, in 1998).

### The First UK National Household Psychiatric Survey, 1993

12,000 adults aged 16–64 were selected from a representative sample of 15,000 households in Great Britain, and over 10,000 interviews achieved, a response rate of 80% [5]. Trained lay interviewers used the Clinical Interview Schedule – Revised (CIS-R); scores were converted into ICD-10 diagnoses; 12 or more was taken to indicate 'likely to have a neurotic disorder'. A separate alcohol and drug schedule was used, and people with 'possible psychosis' were identified for a SCAN interview with a clinician. Occupational social class, income, material standard of living, housing status, education and employment were recorded [16].

An occupational social class gradient for women largely disappeared with adjustment for more precise indicators of social disadvantage. For men, the highest social class had about half the positive scores of other classes, unchanged by adjustment [17]. Unemployment was associated with higher positive scores, and was the factor most strongly associated with symptom prevalence in men and women, while low material standard of living and poor education had the highest rates of probable neurosis. However, the association with education disappeared when adjusted for other socio-demographic variables.

### The Health and Life-style Survey (HLS), 1984–85 and 1991–92

9,003 residents of Great Britain aged 18 years or over, were interviewed, and 82.4% of these examined by a nurse. 6,572 GHQ-30 questionnaires were completed, a score of 5 or more being considered positive; scores were continuously varied for both men and women [18]. Data on occupation of head of household, income, housing tenure and education were recorded. Though not designed as a cohort study, after 7 years 5,352 people (59% of the original sample), were traced and re-interviewed [18]. GHQ scores related to occupational social class showed no consistent pattern. Unemployment was clearly related to high scores in 1984/85, but not in 1991/92.

Of special interest was the finding that positive scores in 1984/85 were associated with significantly increased all-cause mortality after seven years, even after adjusting for age, sex, social class, smoking behaviour, and limiting long-standing illness, and after removing 'un-natural' deaths which might have been specifically related to psychiatric disorder. There was an approximately linear relationship between the risk of dying and the number of symptoms on the GHQ-30, especially for men [19].

### The British Household Panel Survey (BHPS), 1991–92

Of 7,488 British households selected, 5,511 were contacted, involving 10,264 individuals aged 16 and over, of which 9,064, 88% of subjects, completed the GHQ-12. They were followed up a year later. A score of 3 or more was considered 'positive'. Occupation of subject, parents, and head of household were recorded, together with employment data [20]. An indicator of material standard of living combined income, and elements of housing and possessions.

The results gave a gradient with occupational social class (subject or head of household, but not parents), which disappeared in men up to age 55 after adjusting for material standard of living, but was still true for women of all ages [21]. Material standard of living was strongly associated with high frequency of GHQ positives (3+), but possibly only maintainance, not onset of common mental disorders. 'Subjective financial strain', (one question with three possible answers), was correlated with onset of symptoms [22]. Physical illness was associated with GHQ-12 positives. Using also one-year follow-up data, unemployment was also associated with maintenance but not onset of symptoms, which diminished in those gaining employment in the year, and increased in those losing employment in the year, unless for looking after the family or retirement. Scores also decreased during the year for those marrying, and increased for those divorcing or separating.

### The Netherlands Mental Health Survey & Incidence Study (NEMESIS), 1996

7,147 individuals aged 18–64 (64.2%) were interviewed from 11,140 eligible households using the CIDI (and SCID if psychosis was indicated). 43.6% of those refusing the CIDI completed the GHQ-12. Refusers proved to have similar mental health profiles to responders. Family income, average net income per person, employment status, and years of education were recorded [23]. 5,618 adults were interviewed after one year, 79.4% of the cohort [24].

The three commonest disorders, anxiety, depression and alcohol were often present together. Men had more alcohol and other drug disorders; women had more anxiety and depressive disorders. Very poor education, low income, and 'non-employment' were associated with both mood and anxiety disorders [23]. The one-year follow-up showed unemployment associated with the common mental disorders [25].

### The National German Health Survey, 1999

The German Health Survey of 1999 used the CIDI and DSM IV to identify 'cases' in a realised sample of 4,181. Data for 12-month prevalence of 'any mood disorder' and 'any anxiety disorder', relating fairly closely to the 'common mental disorders' are available [11,12]. Prevalence rates are very similar to similar surveys elsewhere. 12-month prevalence was analysed for level of school achievement, employment, and an index of 'social class' combining education, income and job status. In each case high prevalences were found in the more disadvantaged groups (Table 4)

**Table 4:** German National Health Survey 1999, Mental Health Supplement [12].

12 month prevalence:						
	Any mood disorder			Any anxiety disorder		
	%w	OR	CI	%w	OR	CI
**Education**						
Hauptschule (2nd y school)	13.2	1.0		15.4	1.0	
Mittlere Reife (= 'GCSE')	12.2	0.9	0.7–1.1	14.8	0.9	0.7–1.1
Abitur (= 'A levels')	9.5	0.7	0.5–0.9	11.3	0.7	0.5–0.9
						
**Employment**						
FT employed		1.0			1.0	
Unemployed	20.0	2.3	1.6–3.2	23.2	2.2	1.6–3.0
						
**Social Class **(an index combining education, income, and current job status)						
Low	16.4	1.0		18.6	1.0	
Medium	12.0	0.7	0.6–0.9	14.4	0.8	0.6–0.9
High	8.8	0.5	0.4–0.7	11.3	0.6	0.4–0.8

People with Abitur level education had less illness, just significant at the 0.05 level. The unemployed had significantly more illness than the full-time employed. People in medium and high class groups had significantly less illness than those in the low class group (this indicator incorporates education and income with occupation). These results harmonise closely with the overall results of the Inequalities Review described above.

### Summary of the six major European studies (Table 5)

**Table 5:** 'Positive' associations with less privileged social status and the common mental disorders in European surveys (adapted from [6])

	European Surveys	Education	Employment status	Income and material standard of living	Occupational social status
1	HSE 1993+	-	-	positive association for income progressive for both men and women 1998	No clear distribution for either men or women
2	UK Psych Survey 1993	Positive for no qualifications or least years of education for both men and women	Positive for unemployed in both men and women	Positive for income, housing type/tenure, and car ownership	Positive for women (SC I+II compared to SC IV+V); positive for men (SC I compared to all other classes)
3a	HLS 1984/85	-	Positive for unemployment in men in both age groups	-	No clear social class distribution
3b	HLS 1991/92	-	No clear relationship	-	No clear social class distribution
4	BHPS 1991/92	-	Unemployment associated with maintenance, not onset in 1-year follow-up; symptoms reduced on gaining employment (men and women combined)	Positive for low income, 'poverty index', and index of material standard of living (men and women combined)	Positive association for both men and women
5	NEMESIS 1996	Positive for least education (men and women combined)	Positive for unemployment (men and women combined)	Positive for income (men and women combined)	-
6	GHS 1999	Just positive for lowest qualifications (men and women combined)	Positive for unemployment (men and women combined)	-	Positive for SC index combining education, income & job status (men and women combined)

#### Other surveys

Although the Survey of Surveys found few directly comparable studies, some results can be compared in their internal relationships, in the same manner as the literature review described above, and three studies provide data on markers of social disadvantage. These studies would not have fulfilled the strict inclusion criteria for that review, neither have they been subject to the validating processes undertaken in that review. The results should, therefore, be treated with caution.

The Northern Ireland Survey of 1997 [26] used the GHQ-12 with a cut-off point of 3 or more indicating a 'possible case'. Using the UK Occupational Social Classification, lower groups (classes III manual – V) showed higher prevalences than higher groups (I – III non-manual) for both men and women except in the youngest age group. The largest difference was in women aged 45–64. Over age 65, social class differences were very small. Using more detailed Socio-Economic Groups (SEGs), prevalences were progressively higher with lower SEG. Using an education marker of 'some formal qualification' compared with 'no formal qualification', the former had markedly lower prevalence in women, especially young women, but differences were very small in men. People who owned their own house had lower prevalence than those who rented, and those who had access to a car had less than those who did not. Those on lower incomes had higher prevalence than those on higher incomes: twice the rate at age 16–44; twice the rate at age 45–64 in men, three times the rate in women.

These results, though from a smaller survey, are similar to the general results from the large-scale British surveys.

A survey in Belgium in 1997 [27] used the GHQ-12 with a cut-off point of 2 or more. There was no clear detailed pattern in relation to educational level, but 'primary school only' had higher results than all those 'more than primary' combined. A separately recorded 'depression score' (for the previous 12 months) did show markedly less positive scores with better education.

A study of two regions in France, Basse Normandy and Ile de France [28], found that being unemployed was associated with significantly more depression than other employment groups, but education was a mixed and equivocal picture.

If we add to Table 2 the results of these studies and the available findings of the German Health Survey of 1999, (acknowledging the provisional nature of some of the data) we get an expanded Table 6:

**Table 6:** Expanded number of studies reporting associations with higher rates of the common mental disorders, by indicators of less privileged social position.

		*Less education*	*Unemployment*	*Lower income or material circumstances*	*Low social status*
Number of studies reporting associations	Total reporting	9	9	7	8***
Positive	Men & women separately	5**	5*	3	4
association	Men & women combined (separate data not given)	2	3	4	1
	Total positive	7**	8*	7	5
No clear association		2	1	0	3
Inverse association		0	0	0	0

Note: *one study positive only for men; women equivocal; **one study positive only for women; equivocal for men; *** the German 'social class' incorporated education and income as well as occupation.

This adds a little extra weight to the major review without altering the general picture. It is still most notable that no study has given an inverse association between the three markers of social disadvantage and the prevalence of the common mental disorders.

## Initial results from ESEMeD

The European Study of the Epidemiology of Mental Disorders (ESEMeD/MHEDEA 2000) was a comparison of cross-sectional samples of the non-institutionalised population aged 18 years or more, in six countries: Belgium, France, Germany, Italy, the Netherlands and Spain [29]. Different private companies were contracted to undertake the survey in each country. Trained interviewers used a computer-assisted personal interview (CAPI) including the most recent version of the Composite International Diagnostic Interview (CIDI 2000) to assess the presence of mental disorders in face-to face interviews in people's own homes. The total combined sample chosen was 38,015 people, of which 19,706 were interviewed. Response rates varied from 42.1% in France to 71.9% in Spain, giving an overall response rate of 55% [30,31].

We have examined data made available from the ESEMeD study. These are in the form of distributions of odds ratios (ORs) for associations in individual subjects between various social indicators and psychiatric disorder in the 12 months previous to interview. Unemployment data (having a job against not having a job) are the most relevant to inequality analyses; living alone (against not living alone) could possibly have a bearing; receiving Government Assistance (against receiving none) could be very relevant, but the data are not considered reliable by the researchers. Interpretations of data are generally prejudiced by low response rates.

As regards unemployment, all ORs were positive for 'any psychiatric disorder in the previous 12-months', but two of them were not significant; the highest OR (2.49) was Germany. For 'any mood disorder in the previous 12 months', the OR for The Netherlands was negative but not significant; all others were positive and significant at the 5% level; the highest being Germany (OR 5.42). For 'any anxiety disorder in the previous 12 months', all ORs were positive but only two were significant – Germany (OR 1.72) and Italy (1.70). For 'any alcohol disorder in the previous 12 months'. five countries gave positive ORs but only Germany was significant (OR 4.47).

In general, these unemployment results indicate the expected association in individual subjects with psychiatric disorder, but interpretation of the difference between results for different country samples will have to await further analysis. In particular, it will be interesting to see if any light can be thrown upon the tendency of the figures for Germany to be consistently much higher than other countries. The explanation may lie in differences in sampling and interviewing, as each country organised these through different agencies.

The results for living alone offer no particular interest. Of 4 analyses (any disorder; mood disorder; anxiety disorder; alcohol disorder, in the 12 months prior to interview) including all 6 countries, only one result was statistically significant at the 5% level – Germany (OR 1.71 for any 12-month disorder)

For receiving Government Assistance, most analyses were not signifcant. For 'any 12-month disorder', only Germany (OR 1.36) and Italy (OR 1.37) gave significant results. For 'any 12-month mood disorder', only Italy (OR 1.26) and The Netherlands (OR 1.15) gave significant results. For 'any 12-month anxiety disorder', no result was significant. Especially in the light of the doubts of the researchers about the reliability of these data, no interpretation can be offered.

## Estimates of size of effect and of relative risk

While there is clearly broad consistency in the findings, these analyses tell us nothing of the size of effect. Examination of the odds ratios available from the studies under consideration shows that only rarely did they exceed 2, which indicates a doubling of the risk in less privileged groups for the common mental disorders, compared with more privileged groups [6]. ORs and 95% confidence intervals can be summarised for the different markers.

Education:

• In the 1993 British Psychiatric Morbidity Survey (sample aged 16–64), men with no educational qualifications had an OR of 1.29 (1.03–1.62), and women had an OR of 1.26 (1.06–1.49) for recent neurotic disorder, compared to those with A level qualifications (university entrance);

• In the 1996 Netherlands national survey, (sample aged 18–64), people with 0–11 years of education had an OR of 1.55 (1.22–1.98) for mood disorders, compared to people with 16 or more years of education.

Because data are so few from European studies, it is worth adding:

• In the 1990–92 USA NCS (sample aged 15–54), people with 0–11 years of education had an OR of 1.79 (1.31–2.43) for 'any affective disorder', and an OR of 2.82 (2.26–3.51) for 'any anxiety disorder' in the previous 12 months, compared to those with 16 or more years of education.

• In the 1997 Australian National Survey, people who did not complete secondary school had an OR of 1.53 for affective disorders compared to people with post-school qualifications. The sample included all aged 18 and over, so these results will be confounded by age.

Employment.

• In the 1991–92 UK BHPS, the unemployed had an OR of 1,54 (1.13–2.10) for the maintainance of GHQ-12 'case-ness' (scores of 3 or more at both base-line and one-year follow-up), compared to employed people. The sample was all aged 16 and over, so the results will be confounded by age.

• In the 1993 British Psychiatric Morbidity Survey (sample aged 16–64), people who were unemployed had an OR of 2.59 (2.17–3.10) for recent neurotic disorder, compared to people in full-time employment.

• In the 1996 Netherlands national survey, (sample aged 18–64), ORs of 4.3 (3.24–5.72) for mood disorders, and 2.23 (1.70–2.91) for anxiety disorders were reported for a mixed group of 'disabled and unemployed', compared with people who were employed. This strange grouping prejudices interpretation; disabled people may well have higher rates of mood and anxiety disorders unrelated to employment per se.

Also worth noting:

• In the 1990–92 USA NCS (sample aged 15–54), ORs of 2.2 (1.6–2.9) for 'any affective disorder', and 2.1 (1.6–2.8) for 'any anxiety disorder' (both life-time prevalence), were reported for people not working and neither 'home-makers' or 'students', compared with people who were working.

• In the 1997 Australian National Survey (sample 18 and over), the long-term unemployed (12 months or more) had ORs of 2.4 (1.4–4.3) for 'any affective disorder', and 2.8 (1.6–5.0) for 'any anxiety disorder' compared to employed people. Analysis excluded those 'not in the labour force'.

Income or material standard of living.

• In the 1991–92 UK BHPS (sample16 and over), comparing household income in quintiles, the middle three fifths had more GHQ-12 'positives' (scores of 3 or more), OR 1.16 (1.0–1.34), and the lowest fifth had far more GHQ-12 'positives', OR 1.45 (1.21–1.74) than the highest fifth.

• In the 1993 British Psychiatric Morbidity Survey (sample aged 16–64), people renting their homes had an OR of 2.17 (1.79–2.64) for men, and 1.71 (1.48–1.98) for women, for recent neurotic disorder, compared to people who owned their own homes.

• In the 1998 Health Survey of England (sample aged 16 and over), ORs for the lowest quintile of equivalised household income was 1.53 (1.12–2.09) for men and 1.11 (0.87–1.41 – not significant) for women, for GHQ 'caseness' (scores of 4 or more), compared to the highest quintile.

• In the 1996 Netherlands national survey (sample aged 18–64), ORs of 1.56 (1.20–2.03) for mood disorders, and 1.77 (1.43–2.21) for anxiety disorders, were reported for the lowest income quartile compared to the highest income quartile.

In addition, we might note that:

• In the 1990–92 USA NCS (sample aged 15–54), ORs of 1.73 (1.29–2.32) for 'any affective disorder', and 2.12 (1.63–2.77) for 'any anxiety disorder' (both 12-month prevalence), were reported for people earning $0–$19000 a year compared to people earning $70,000 or more.

## Discussion

In general we might say that the odds against disadvantaged groups are undoubted, but modest, the increased risk being generally between one and a half and two times that for the least disadvantaged groups. However, it should be remembered that these are all rather crude measures of ambiguous phenomena; there is nothing subtle or precise in population surveys of psychiatric illness, although the situation is much better than twenty years ago and is improving still.

We must make do with what we have, and recognise that the conclusions of the recent work described in this paper, whilst undramatic, represent a real advance on previous knowledge. There can be no doubt now that disadvantaged groups in European populations experience more anxiety and depression, measurable on standard instruments and representing significant suffering for individuals, and serious loss of production and social function, with important consequences for children, communities and work-places. We can begin to define populations at risk, though this will still be rather generalised.

The scientific literature from major population studies currently permits very little detailed comparitive analysis of risk factors other than the three presented above, education, employment, and income/material standard of living, which can be measured in fairly similar ways in all western societies. Social Class or Socio-Economic Group can only be a proxy for these, and, no doubt, other more precise and tangible markers of social position and social experience. We now need focussed investigations into causative factors and possible means of prevention, and evaluations of means of relieving suffering and improving function.

The evidence drawn from cross-sectional studies, however large, cannot determine the direction of causation, which is, no doubt, complex, and not all one way. Cohort evidence has the potential to help here, but the little available evidence is fragmentary and supports only tentative conclusions [5]. In general it appears that higher rates of disorder in adulthood are associated with multiple disadvantage in childhood, including parental divorce and economic hardship, and parental psychiatric illness. These causative factors are generally also associated with social disadvantage.

The excess of the common mental disorders in disadvantaged people is well enough established to justify health policy initiatives to ensure that access to effective diagnosis and treatment is improved, especially at the primary health care level, and especially in communities with high levels of social disadvantage. A wide range of treatment strategies should be available, including, where appropriate, drugs, counselling and other therapies, and social interventions to improve disadvantageous situations. Concurrent physical illness must also be addressed in the total treatment package. Interventions need to be properly evaluated.

Research relating mental ill health to social disadvantage has already produced a wealth of useful evidence, but general conclusions useful to policy makers are to some extent prejudiced by incompatible methods, measures and analyses. Standardising and validating a small range of instruments and indicators, and closer collaboration between researchers, especially across the EU, would both facilitate and economise on future studies. But, in the reality of many studies already performed, there is also need for better methods of synthesising disparate findings of this kind [2].

Large scale longitudinal studies are especially needed if the complexities of cause are ever to be teased out. There are also continuing opportunities for exploiting already existing large scale data bases. Little in the literature considered above addresses issues of cultures and sub-cultures, and their impact on the mental health and mental health risks of individuals. Communal, societal influences on experience, behaviour and health, as well as individual actions and attitudes need to inform both research and political action.

## Conclusion

People of lower socio-economic status, however measured, are disadvantaged, and this includes higher frequencies of the conditions now called the 'common mental disorders' (mostly non-psychotic depression and anxiety, either separately or together). In European and similar developed populations, relatively high frequencies are associated with poor education, material disadvantage and unemployment. Their large contribution to morbidity and disability, and the social consequences in working age adults would justify substantial priority being given to addressing mental health inequalities, and deprivation in general, within national and European social and economic policy.

But disadvantaged people also tend to live in communities and cultures that are disadvantaged by noxious environments, poor human services, high levels of smoking, drinking, drug taking, and violence. These are almost certainly causally associated with high levels of psychiatric morbidity also found, possibly mediated or enhanced by poor education, low incomes and low status work. These factors may affect duration as well as onset and thus increase prevalence in populations.

However, there are well known policy implications relating to social exclusion and deleterious social environments; it does not need population surveys to show that serious poverty, deprivation, environmental degradation and social stress should be high on the political agenda; it is a matter of equity, justice and human rights.
